# Successful endoscopic closure of a large esophageal perforation using the purse-string technique

**DOI:** 10.1055/a-2078-0761

**Published:** 2023-05-10

**Authors:** Michael Lajin, Naser Khan, Fateh Bazerbachi

**Affiliations:** 1 Sharp Healthcare, San Diego, California, USA; 2 Javon Bea Hospital-Rockton, Rockford, Illinois, USA; 3 CentraCare Health System, St. Cloud, Minnesota, USA

Endoscopy has been increasingly utilized to manage esophageal perforations 1 2 3 .

We present in this case a successful endoscopic closure of a large esophageal perforation using the purse-string technique 4 5 ( Video 1 ).

**Video 1** Endoscopic closure of a large esophageal perforation.


A 79-year-old woman with multiple comorbidities underwent a savary dilation of esophageal stricture at an outside institution. She presented with septic shock, lactic acidosis, and respiratory failure.

A chest x-ray revealed pneumomediastinum and a right-sided pneumothorax. A chest tube was inserted. She was resuscitated with intravenous fluids, bicarbonate, antibiotics, and three vasopressors. A chest computed tomography (CT) revealed pneumomediastinum and improved pneumothorax after chest tube insertion without mediastinal fluid collections. She was deemed a poor candidate for surgery.

Endoscopy revealed a large mid-esophageal perforation ( Fig. 1 ). Endoscopic suturing was challenging due to the narrow space. After a running suture was torn, we decided to switch to the purse-string technique.

**Fig. 1:**
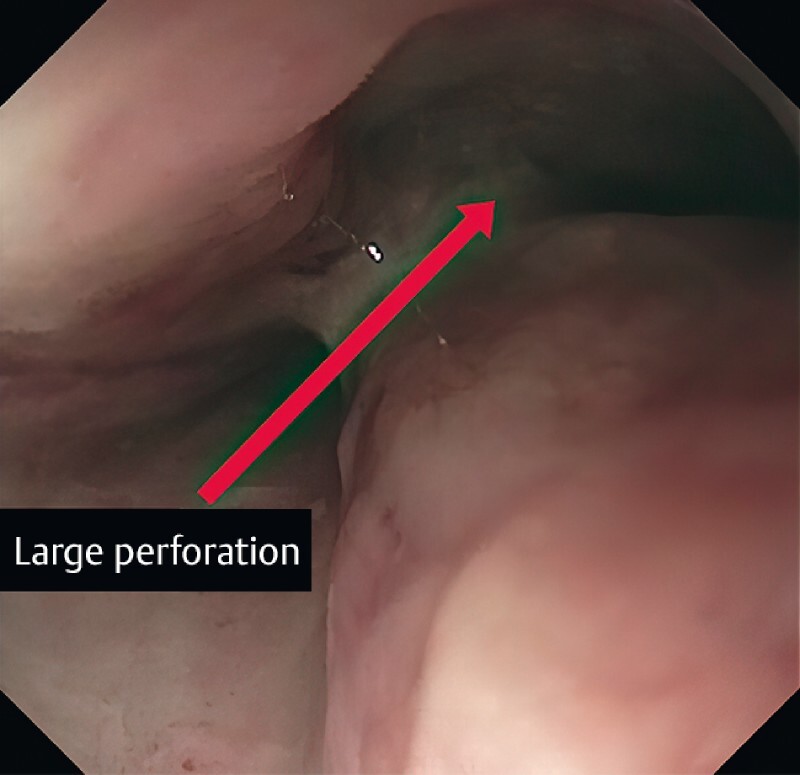
A large full-thickness esophageal perforation (red arrow).

An endoloop catheter and a clip were advanced through the two channels of a double-channel endoscope. The endoloop was fixed to the margins of the perforation by clips. The endoloop was then tightened approximating the margins of the defect ( Fig. 2 ) and deployed. A residual defect was seen proximally and a second endoloop was tightened and deployed similarly.

**Fig. 2:**
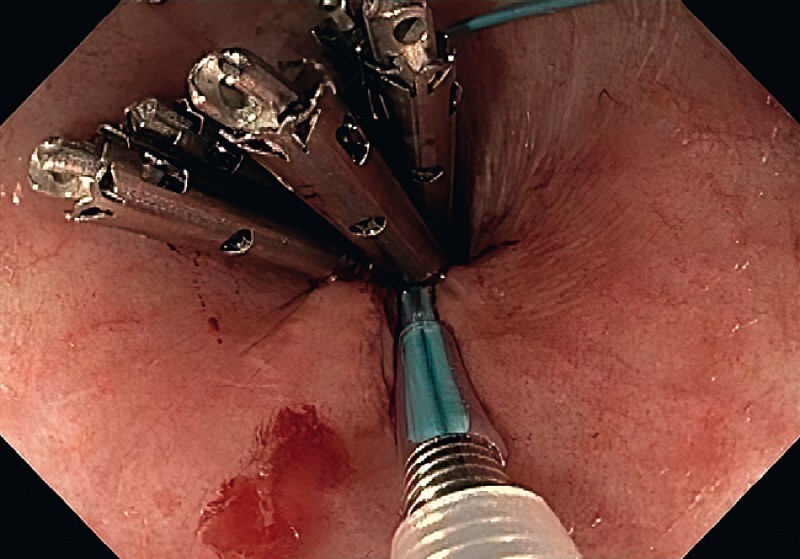
Endoscopic view of the string-purse technique after tightening the endoloop.

A through-the-scope esophageal stent (18 mm × 149 mm) was deployed covering the site of perforation ( Fig. 3 ). The proximal end of the stent was sutured to the esophageal wall to prevent migration.

**Fig. 3:**
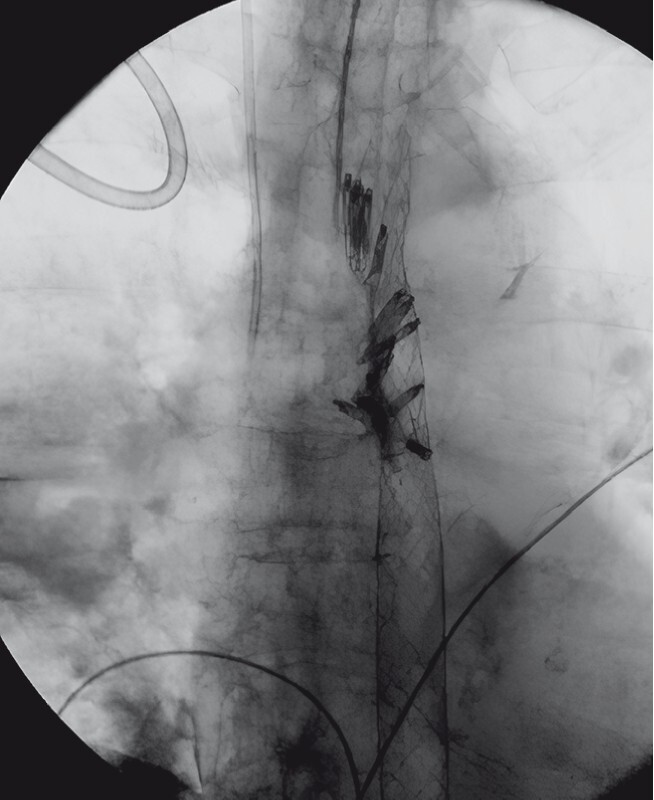
A fully covered esophageal stent deployed after the primary closure of the defect.

Five days after the procedure, she was extubated and weaned off vasopressors. Two weeks after the procedure, chest CT showed a remarkable decrease in the pneumomediastinum.

She was weaned off oxygen, and the chest tube was removed. Enteral feeding was started through a jejunal extension of a preexisting gastrostomy tube.

Three weeks after the procedure, an endoscopy was performed. The esophageal stent was retrieved. The perforation site had healed ( Fig. 4 ). Contrast injection demonstrated complete sealing of the esophageal wall ( Fig. 5 ).

**Fig. 4:**
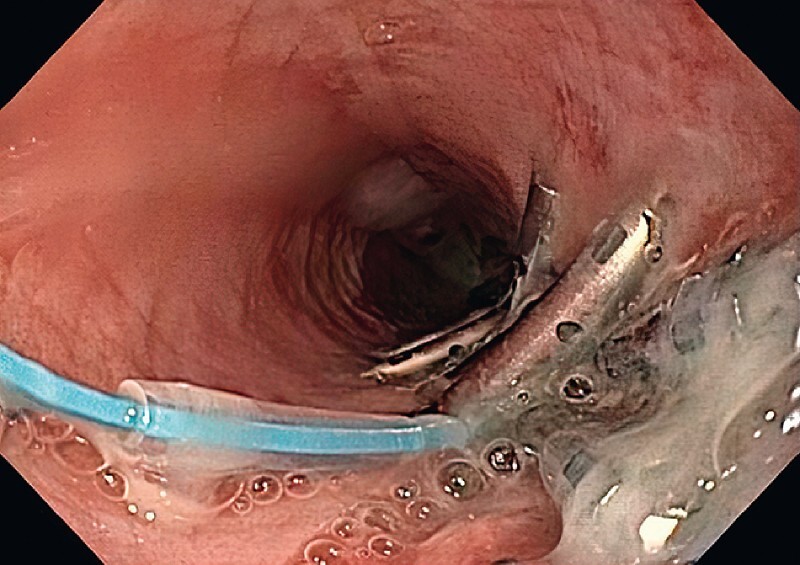
Endoscopic view after 3 weeks, demonstrating closure of the defect.

**Fig. 5:**
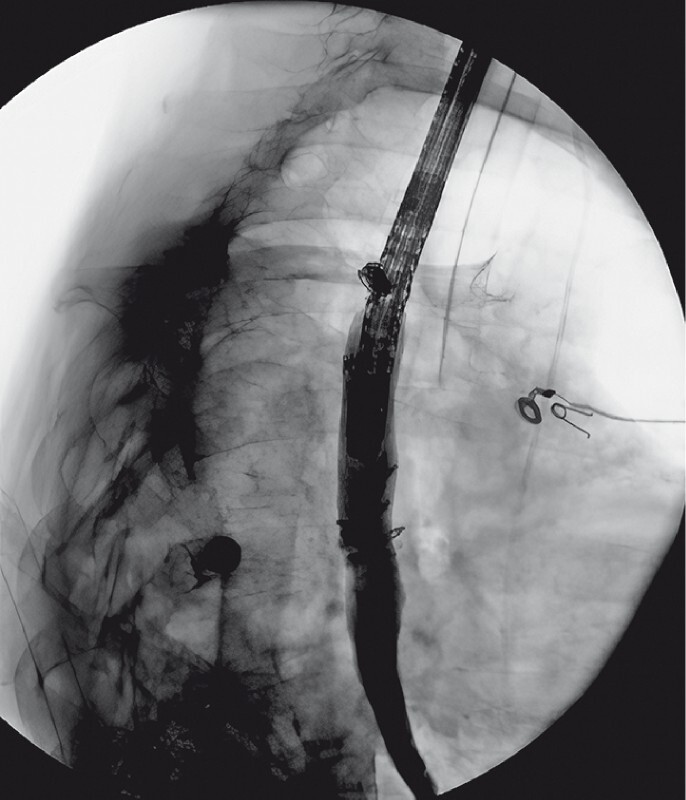
Contrast injection after esophageal stent removal showing complete sealing of the esophageal wall with no extravasation of contrast.

Endoscopy_UCTN_Code_TTT_1AO_2AI

## Notes

Endoscopy E-Videos

https://eref.thieme.de/e-videos
*E-Videos* is an open access online section of the journal *Endoscopy* , reporting on interesting cases and new techniques in gastroenterological endoscopy. All papers include a high-quality video and are published with a Creative Commons CC-BY license. Endoscopy E-Videos qualify for HINARI discounts and waivers and eligibility is automatically checked during the submission process. We grant 100% waivers to articles whose corresponding authors are based in Group A countries and 50% waivers to those who are based in Group B countries as classified by Research4Life (see: https://www.research4life.org/access/eligibility/ ). This section has its own submission website at https://mc.manuscriptcentral.com/e-videos
